# The risk of hydraulic fracturing on public health in the UK and the UK’s fracking legislation

**DOI:** 10.1186/s12302-015-0059-0

**Published:** 2015-10-30

**Authors:** Elisabeth Reap

**Affiliations:** Environment Department, University of York, Heslington, York, UK

**Keywords:** Hydraulic fracturing, Fracking, Air emissions, Public health, Risk, Uncertainty, Precautionary principle, UK

## Abstract

**Background:**

Hydraulic fracturing to extract natural gas from shale rock is a new, rapidly expanding industry in the United States (US). However, there is concern that these operations could be having large negative impacts such as groundwater contamination, increased air pollution and seismic events. The United Kingdom (UK) is looking at the potential for emulating the success of ‘shale gas’ in the US. Differences in population density and geological conditions mean that the public health impacts recorded in the US cannot be directly extrapolated to the UK. There is limited academic literature available but findings suggest that the UK government is not fully recognising the inherent risks of hydraulic fracturing exposed by this literature. Government reports suggest a reliance on engineering solutions and better practice to overcome problems found in the US when evidence suggests that there are inherent risks and impacts that cannot be eliminated.

**Results:**

This study applies US results to approximate the impact of one exposure pathway, inhalation of hydrocarbons by the public from operational air emissions over the 30 year lifetime of a well and finds that 7.2 extra cancer cases from exposure to air contamination would be expected in the UK if all test sites, approved test sites and test sites awaiting approval as of January 2015 went on to extract gas.

**Conclusions:**

In conclusion, limited assessment of the public health implications of hydraulic fracturing operations is available but the UK government appears to not be applying the precautionary principle to potentially significant legislation.

## Background

Over the past decade, technological advances have led to a dramatic increase in the use of hydraulic fracturing (fracking) to extract natural gas from shale rock in the United States of America (US) [1–4]. Other countries are now considering exploiting their own reserves of shale gas. Some, such as France and Bulgaria have already banned fracking, but others, including the United Kingdom (UK), South Africa and Poland are keen to exploit their resources [3]. There exist only a small number of studies conducted in the US investigating the public health impacts of fracking that can be used to assist the creation of legislation [1, 2, 5–8].

The UK is currently exploring its potential for shale gas extraction and the government has passed legislation that allows fracking operations [3, 9–12]. In January 2015 the UK government voted against suspending all fracking activity while an environmental assessment is carried out. The government did concede some ground; however; fracking is now banned in National Parks and other areas of natural interest. The Scottish government used its devolved power to vote against the national UK government for an indefinite moratorium on fracking in Scotland.

Currently in the UK, there is wide debate on whether the country should exploit its shale gas reserves or not. There are many reasons given for wider exploitation, including regional and national economic growth, increased domestic energy security, increased employment opportunities and prospects and reduced domestic carbon dioxide emissions when natural gas displaces the need for using oil and coal in electricity generation [3, 7, 9–11, 13–15]. Conversely, many arguments are made against fracking, such as concerns about seismic activity, air pollution, groundwater contamination and public health [3, 4, 7, 16–18].

The current UK government supports fracking and predicts that it will have huge potential for increasing the supply of domestic natural gas [7, 19]. This attitude has been criticised by McGlade et al. [17] in the UK Energy Research Centres’ latest report which suggests that there is little evidence backing up the government’s claims about the total UK reserve of shale gas and that gas will only be a small part of future energy supplies. Government reports conclude that there is no significant risk to human health posed by shale gas, but this position assumes that best practice is followed at all times and that engineering solutions resolve all public health issues reported in the US [7, 12–14, 16]. For instance, the Public Health England report by Kibble et al. [12] concluded that, based on available information, health risks are low if the industry is properly regulated and best practise is observed [9]. The conclusions drawn by Kibble et al. [12] are not consistent with the literature; however, public health impacts have been associated with US fracking activity and further, the authors do not account for the differences in UK population density and geology [1, 6, 7, 16]. This means that Public Health England is assuming all reported health risks can be overcome by regulation and engineering.

Many scientific studies conclude that the chemicals used or emitted during the fracking process are negatively impacting the health of nearby human populations [1, 2, 5, 8, 20]. The number of people exposed to air emissions from well operations is growing in the US because it is becoming increasingly common for fracking sites to be situated near to where people live and work [1, 5, 6, 8, 20]. Due to the Energy Policy Act of 2005, operators in the US are not obliged to disclose information about the chemicals they use in the fracking process, which means it is hard to predict potential public health impacts [2–4, 7]. Although these chemicals comprise only 1 % of the total fracturing fluid volume, some are known to be toxic even in small doses and cause negative short-term and long-term health effects, such as formaldehyde, diesel and naphthalene; also, there are other chemicals used that we know little about [1, 2, 20]. Natural gas itself is comprised of many chemicals: it is predominantly methane but can also contain alkanes, benzene and other aromatic hydrocarbons [1, 9]. Toxic chemicals, including arsenic and lead, exist naturally in shale rock and they are extracted along with the natural gas which threatens the quality of local groundwater [2].

Groundwater contamination, surface spills of fracturing fluid, air emissions and faults in offsite disposal of contaminated water are all examples of problems that can develop into human exposure pathways [1–3, 5, 13, 21, 22]. For example, the inhalation of hydrocarbons due to air emissions from gas extraction has been reported to cause headaches, fatigue, temporary limb paralysis and unconsciousness depending on the level of exposure [1, 5]. Air emissions from fracking operations include ozone, volatile organic compounds (VOCs), particulate matter and nitrous oxides, all of which are known to negatively impact health [2, 6, 13, 20]. Exposure to harmful chemicals due to fracking activity cannot be eliminated through regulation as there are technological and economic limitations to the treatment of emissions into the air, into groundwater and from waste; thus fracking is an inherently risky process in terms of human health [3, 5].

A study by McKenzie et al. [1] found that residents who live closer to wells have a higher risk (unquantified) of experiencing negative respiratory, neurological and reproductive health impacts from air emissions caused by fracking operations. Residents living within half a mile of well sites were found to have an excess cancer risk of 10 in a million averaged over a lifetime of 70 years from a chronic 30-year exposure to a fracking well [1]. The risk to residents living over half a mile away is six in a million; therefore, residents who live closer to well sites have a cancer risk 1.67 times higher than those living further away [1]. For comparison, research suggests that those exposed to high levels of air pollution rather than low levels are 1.5 times more likely to have cancer, and workplace environmental exposure to tobacco smoke increases the risk of lung cancer in adult women and men by 1.15 and 1.28, respectively [23]. The inhalation of benzene, a constituent of natural gas, was found to be the largest contributor to this risk in the study by McKenzie et al. [1, 5]. The results of this study will later feature in analysis of the potential impact on UK public health of the exposure pathway for inhaled hydrocarbons emitted by fracking operations.

Bunch et al. [6] also studied the health impacts of fracking but focused on VOCs. The study concluded that increased exposure to VOCs with proximity to fracking operations is not a health concern [6]. However, the study neglected to recognise that workers and residents are assigned different levels of acceptable risk in the US. Workers are assigned a higher acceptable risk level (1 in 10,000) than residents (1 in 1,000,000) because they spend time in close proximity to the potentially harmful exposure pathways by choice, rather than by circumstance [24]. This is an example of a false negative result which can ill-inform policymakers. Recognising this difference would mean the study would have concluded that in some regions the general public were exposed to an unacceptable risk from VOCs by US standards.

Below, the future impact that fracking operations may have in the UK is estimated and discussed. This includes an investigation into the extent to which the UK government is utilising existing scientific evidence when creating new fracking legislation and an estimation of the extra health costs to the UK using results based on the effects of air emissions of hydrocarbons in the US.

## Results and discussion

Results of the calculations performed using the data sourced for this report and McKenzie et al.’s [1] work find that if all sites that have drilled wells for fracking, that have been approved for fracking and that are under consideration for approval as of January 2015 commence fracking operations (scenario 3), then the UK will experience approximately seven extra cancer cases caused by the inhalation of hydrocarbons from fracking operations (Table 1*)*. The temporal unit of these cancer cases assumes an operational well lifetime of 30 years averaged over a default human lifetime of 70 years [1]. These calculations do not include an assessment of non-cancerous health effects or cancerous health effects caused by other pathways of exposure and other chemicals. These results express an example of one of the potential impacts that fracking operations could have on the UK. However, as discussed previously and below, results for US data cannot be directly applied to the UK, so these calculations are intended to only illustrate an idea of the potential public health impact that one exposure pathway may cause, not to be used as concrete evidence of possible future public health problems.

**Table 1:** Summary of estimations made to illustrate the potential impact of fracking operations in the UK

Scenario	Description	Additional cancer cases
1	Includes all sites that have drilled wells for fracking use. Excludes sites where interest in fracking has been withdrawn	2.7
2	Includes previous + all sites that have approved planning permission to drill wells	6.7
3	Includes previous + all sites that are under planning consideration	7.2

Description of three future potential fracking scenarios in the UK and the estimated additional cancer cases these scenarios may cause in the public. The additional cancer cases for each scenario were estimated using data from McKenzie et al. [1], the 2011 census in England, Wales and Northern Ireland and information from planning applications and fracking operators

The UK has the opportunity to review existing scientific evidence from the US before passing legislation on fracking. However, there are key differences between the two countries, which mean that US findings cannot be directly applied to the UK [13]. The UK is almost ten times more densely populated than the US, averages of 265 and 27 people km^−2^, respectively [25]. Additionally, shale gas resources are spread much more thinly in the UK and geological conditions are not the same as US conditions, where there is also geological variability between different shale gas production sites [9, 13, 14, 26, 27]. One example of differences in geological conditions at production sites which may alter the ability to extrapolate results is that shales in the US are exploited only if they have low proportions (<50 %) of clay minerals ‘to allow for successful fracture stimulation’ [27] but the UK is known to have medium to high proportions of clay minerals [26, 27]. Another example is that there is no evidence of overpressure in potential UK shales which means that well production rates are likely to be much lower than quoted US figures [14]. Direct comparison between the two countries without caveats is inadvisable as US data cannot be accurately extrapolated to fit UK scenarios and, therefore, UK policymaking should not be based solely on US figures.

In reviewing US evidence, however, UK government reports conclude that inherent risks found in US fracking operations can be overcome by regulation and engineering solutions. Not all fracking risks are due to bad regulation and procedure in the US: accidental spills, operational emissions and cement or well casing failure are not entirely unavoidable; these risks are inherent with the fracking process [4, 16]. For example, between 2010 and 2012 in Pennsylvania, US, between six and seven per cent of well casings failed due to compromised structural integrity [28]. The UK regulations claim to minimise the risks derived from fracking; however, in England and Wales there is no set minimum distance between industrial activity and populated areas [9, 10, 19]. This ignores evidence that geographical distance is a key variable affecting cancerous and non-cancerous health effects in residents near fracking sites [1, 8, 20].

Assuming the precautionary principle, protection of public health should take precedence over enhancing its welfare through increased investment and employment opportunities [3, 4]. Uncertainty can inspire sometimes dangerous decisions to be made under false negative and, less commonly, false positive results [3, 29]. De Melo-Martin et al. [3] argue that this uncertainty means the potential benefits of a best-case scenario for fracking do not outweigh the potential costs of a worst-case scenario.

As previously discussed, potential exposure pathways and, therefore, health effects resulting from fracking activity can be reduced but never fully eradicated. The precautionary principle should be applied to avoid false negative results and conclusions, such as Bunch et al. [6], becoming the premise of legislation. The UK is already applying ideas that improve upon the US framework in the fracking industry, such as compulsory disclosure of chemicals in use, but there are also many areas where legislation can be improved, for example setting a minimum distance between residential areas and fracking operations [9]. Medical professionals should be made aware of the effect of fracking on health. Continued monitoring and research should be carried out to reduce uncertainty and improve regulation of the fracking industry [5]. However, the safest approach with regard to public health would be to dismiss fracking as a viable option and promote energy technologies that are known to have less of an impact on human health [3].

## Conclusions

The academic literature related to fracking and public health is very limited. There are uncertainties and difficulties in applying results from the US to the UK which means that precaution should be taken when developing legislation and regulation of the fracking industry for the UK from US data. Some studies have found evidence that fracking is having a significant negative impact on local public health in the US [1, 6, 8]. Estimates using assumptions and US cancer risk data alone have suggested that if all sites that have been currently test drilled, approved for test drilling and under consideration for approval go on to exploit shale gas reserves, then approximately 7.2 excess public cancer cases will be caused by fracking operations over a default 70-year human lifetime if the wells are in operation for 30 years. This work considers the potential effect on public health of inhaling airborne hydrocarbons created by fracking operations but does not discuss other possible impacts of fracking activity such as the contamination of groundwater and seismic activity. The UK has acknowledged and developed upon issues seen in US regulation of fracking. However, too much emphasis is being placed on the suggestion that engineering solutions and legislation can neutralise the problems associated with fracking. It is not being acknowledged that there are inherent risks in the industry. Therefore, the conclusion can be made that the UK government is not fully recognising evidence in the literature when creating fracking legislation and regulations.

## Methods

Data from the 2011 Census in England, Wales and Northern Ireland at the lowest area level were used to estimate the number of people residing within a ½ mile (0.8 km) and 10-mile (16 km) radius of sites that have experienced exploratory fracking activity, sites that have been approved for exploratory fracking and sites that are under consideration as of January 2015 [30, 31]. Information on individual fracking locations was sourced from local authority planning applications and the websites of the operator companies. Unfortunately, as there are no comprehensive data set some sites may have been missed. For example, it has been widely reported that iGas have drilled test wells in England but exact locational information could not be sourced [32]. As previously mentioned, there is no current potential for fracking activity in Scotland so it is not included in these scenarios. Also, sites that have previously been approved for test drilling and are located within the boundaries of National Parks have been discounted due to the new regulations mentioned previously. Finally, some sites have been drilled for exploration purposes, but the operator has then said that no further action will be taken to pursue a fracking licence, so these sites were also excluded from the analysis. Table 2 describes all the sites included in this analysis.

**Table 2:** Details of all potential fracking sites in the UK as of January 2015

Operator	Status	Location and site name	Civil parish or equivalent	Population density (people per hectare)	People in ½ mile radius	People between ½ and 10-mile radius
Rathlin energy	Drilled	North Yorkshire—Crawberry Hill	Border between Bishop Burton and Walkington	1	203.4	81,163.9
Rathlin energy	Drilled	North Yorkshire—High Fosham Cottage	Burton Constable	0.1	20.3	8116.4
Third energy	Drilled	North Yorkshire—Kirby Misperton 1 (East)	Kirby Misperton	0.5	101.7	40,582.0
Cuadrilla resources	Drilled	North West—Becconsall	North Meols	1.3	264.4	105,513.1
Magellan petroleum	Drilled	South East—Horse Hill	Salfords and Sidlow	1.7	345.8	137,978.6
Cuadrilla resources	Drilled	South East—Lower Stumble	Balcombe	0.9	183.1	73,047.5
Cuadrilla resources	Approved	North West—Hale Hall Farm	Treales, Roseacre and Wharles	0.3	61.0	24,349.2
Coastal oil and gas	Approved	Wales—Unit 1 Llandow Industrial Estate	Llandow	0.6	122.0	48,698.3
Cuadrilla resources	Approved	South East—Sugham Farm	Lingfield	5.1	1037.3	413,935.9
Celtique energie	Approved	South East—Wood Barn Farm	West Chiltington	1.9	386.5	154,211.4
Infrastrata PLC	Approved	Northern Ireland—Woodburn Forest	Antrim	0.2	40.7	16,232.8
Rathlin energy	Under consideration	Northern Ireland—49 Ballinlea Road	Antrim	0.2	40.7	16,232.8
Cuadrilla resources	Under consideration	North West—Roseacre Wood	Treales, Roseacre and Wharles	0.3	61.0	24,349.2
Cuadrilla resources	Under consideration	North West—Preston New Road	Westby-with-Plumptons	0.6	122.0	48,698.3

The data from the 2011 Census in England, Wales and Northern Ireland used alongside planning applications and information from fracking operation companies that will be used to calculate the impact of fracking on public health

Three scenarios of potential future fracking activity in the UK have been explored. Scenario 1 assumes that all sites that have been approved for exploratory drilling and have acted upon that approval will continue on to produce shale gas. Scenario 2 includes all sites from scenario 1 and also those sites that have had drilling approval but not yet started operation. This scenario assumes that all these sites are explored and then all the operators apply for and are granted permission to and commence fracking operations to exploit shale gas resources. Scenario 3 includes all sites in scenario 2 and additionally all those that are currently under consideration for permission to drill test wells. This scenario assumes that all those under consideration go on to be fully operational shale gas extraction sites.

Table 1 describes three potential scenarios for future shale gas exploitation in the UK and shows the results of analysis using work by McKenzie et al. [1] to estimate excess cancer cases caused by each scenario. The values of excess lifetime cancer risk were determined using the US Environmental Protection Agency’s methodology and ambient air sample data collected in Garfield County, Colorado, US [1]. The study examined the non-cancerous and cancerous effects of unconventional natural gas wells on local residents and, among other things, produced risk values for cancer in residents who were inhaling hydrocarbons in air emissions from gas operations. McKenzie et al. [1] concluded that further study of this and other pathways of exposure was required in the US due to the nature of their findings. Their work was the only example found where the risk of certain public health effects had been calculated and numerically defined; this is the reason why the results of McKenzie et al.’s [1] work were used as the basis of this assessment. However, the calculations performed using McKenzie et al.’s [1] results must be treated as estimates as the risk values of excess cancer cases from inhalation exposure pathways in the US may not be the same as in the UK. Consequently, the calculations in this paper also take on the assumptions made in McKenzie et al.’s [1] work as well as also assuming that population density is uniform within each Civil Parish of the 2011 Census. A further assumption is that the effects of emissions further than 10 miles from a fracking site are insignificant [8]: the calculations, therefore, assume uniform risk of excess cancer cases between ½ and 10 miles of fracking operations. A breakdown of the calculations is shown in Table 3.

**Table 3:** Calculations of estimated public health impact of fracking in the UK

Site type	Number of people living less than ½ mile away	Risk	Extra cancer cases	Number of people living between ½ and 10 miles away	Risk	Extra cancer cases	Total extra cancer cases
Sites with drilled wells	1118.7	1 × 10^−5^	0.011	446,401.5	1 × 10^−6^	2.678	2.690
Site approved for drilling	1647.54	1 × 10^−5^	0.016	657,427.59	1 × 10^−6^	3.945	3.961
Sites under planning consideration	223.74	1 × 10^−5^	0.002	89,280.29	1 × 10^−6^	0.536	0.538

A breakdown of the calculations used to estimate the number of excess cancer cases that may be caused if fracking operations commence in the UK. The risk values were found from McKenzie et al. [1] and the population numbers are shown in Table 2

## Abbreviations

Fracking: hydraulic fracturing
US: United States of America
UK: United Kingdom
VOCs: volatile organic compounds
